# Heavy‐Atom Tunneling at Room Temperature Revealed by Instanton Theory

**DOI:** 10.1002/anie.2372199

**Published:** 2026-09-23

**Authors:** Debaarjun Mukherjee, Kamil D. Petryczkiewicz, Jeremy O. Richardson

**Affiliations:** ^1^ Department of Chemistry and Applied Biosciences ETH Zürich Zürich Switzerland

**Keywords:** chemical reaction, cubane, diradical, instanton, intersystem crossing, potential energy surface, quantum chemical modeling, quantum tunneling, spin crossover, triplet state

## Abstract

Quantum‐mechanical tunneling enables molecules to react even when their energy is below the activation barrier. Nonetheless, it is not normally expected that the dominant mechanism of chemical reactions relies on tunneling except at very low temperatures, especially when heavy atoms are involved. This assumption is valid for most adiabatic reactions proceeding on a single potential energy surface. However, we argue that heavy‐atom tunneling at room temperature may be more important than previously supposed in the case of nonadiabatic processes, such as spin‐forbidden reactions. In this work, we employ a recently developed computational approach (called nonadiabatic instanton theory) to capture the optimal multidimensional tunneling pathway for the ring closing of various strained organic triplet diradicals to form housane and a cage dione (which is a precursor for cubane). Surprisingly, we find that these reactions are dominated by the tunneling of heavy carbon atoms even at room temperature. This highlights the role of tunneling in spin‐forbidden reactions and may open up the possibility for tunneling control in the synthesis of strained molecules, not just at cryogenic temperatures, but under ordinary laboratory conditions.

## Graphical abstract

Usually, tunneling does not play a significant role in chemical reactions except at low temperature, especially when heavy atoms are involved. However, using nonadiabatic instanton theory we find that heavy‐atom tunneling dominates the reaction mechanism for spin‐forbidden processes in the synthesis of strained organic molecules even at room temperature.

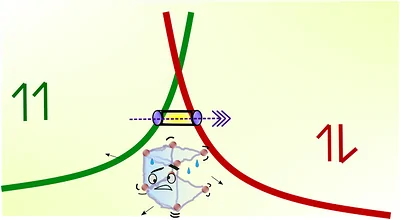

## 1 Introduction

The consequences of molecular tunneling have been discussed since the advent of quantum mechanics [1], with a particular emphasis on its ability to increase the rate of a chemical reaction [2]. There exists a wealth of experimental evidence for hydrogen‐atom tunneling in enzymatic reactions [3, 4], sigmatropic rearrangements [5], hydrogen abstraction [6, 7], organometallic reactions [8] as well as carbene and nitrene chemistry [9, 10, 11, 12]. It has even been proposed that tunneling can be used to control chemical reactivity—referred to as the third reactivity paradigm along with kinetic and thermodynamic control [13, 14, 15]. In addition to H atoms, heavier atoms such as C, N, and O can also tunnel [16, 17]. However, to date, observations of heavy‐atom tunneling have almost exclusively been made at very low temperatures, typically below 50 K, where the reactions take place in cryogenic Argon matrices [18, 19, 20, 21, 22, 23, 24].

These experimental findings can be rationalized by noting that the tunneling probability depends strongly on the particle mass, making tunneling of light atoms such as hydrogen far more prevalent than the tunneling of heavier second‐row elements. In order to quantify the relative importance of tunneling compared to over‐the‐barrier processes for a chemical reaction at a given temperature, we calculate the tunneling factor, κ, which is defined as the ratio of the rate including tunneling effects to the classical result where tunneling is forbidden. For a simple estimate, one can use Bell's formula [2, 25], $\kappa_{\text{Bell}}=\frac{1}{2}\beta \hbar \omega /\sin\left(\frac{1}{2}\beta \hbar \omega \right)$, which is based on a parabolic‐barrier approximation with barrier frequency ω at a temperature defined via $\beta =1/k_{B}T$. A typical barrier frequency for a proton‐transfer reaction is around $1000\;{\mathrm{cm}}^{-1}$ [26, 27, 28], leading to a tunneling factor of $\kappa_{\text{Bell}}=3.58$ at room temperature, meaning that for each over‐the‐barrier process, 2.58 tunneling events also occur, such that tunneling is the dominant reaction mechanism. However, for a similar reaction involving 12‐times heavier carbon atoms, one would expect the barrier frequency to reduce by about a factor of $\sqrt{12}$, leading to a speed‐up of only $\kappa_{\text{Bell}}=1.08$, meaning that tunneling events are rare compared with over‐the‐barrier processes. It is for these reasons that systematic studies of organic reactions could not find a case where the reaction mechanism was dominated by heavy‐atom tunneling at room temperature [29]. The only known exception is the famous case of cyclobutadiene [30]: an autoisomerization which does not break or form bonds.

In this work, we will show that this simple argument, which seems to rule out the importance of heavy‐atom tunneling at room temperature in chemistry [31], is valid only for adiabatic reactions that take place on a single Born–Oppenheimer potential energy surface (PES). However, we expect that quantum tunneling may have a much more pronounced effect in nonadiabatic reactions from a reactant state to a product state, where Bell's formula does not apply, since the cusp at the crossing of the PESs makes the barrier particularly narrow (see graphical abstract) in contrast to the rounded tops of adiabatic barriers. We will therefore investigate nonadiabatic reactions, such as spin‐forbidden processes which transform a triplet reactant into a singlet product. In this case, we find that quantum tunneling can lead to a significant speed‐up of at least a factor of 2 at room temperature, even when heavy atoms such as carbon are involved. In this way, we will demonstrate that, contrary to expectations, heavy‐atom tunneling can dominate the mechanism in reactions relevant for synthetic organic chemistry without requiring cryogenic temperatures.

We will study four illustrative reactions, namely the synthesis of housane (Figure 1, **A3**) [32, 33], [2]‐ladderane (**B3**) [34, 35], cyclobutanecarbaldehyde (**C3**), which is representative of more general photocycloaddition of enones to alkenes [36], and a cage dione (**D3**) that is a slightly simplified version^1^ of a precursor in the synthesis of cubane [37]. These reactions are initiated by photoexcitation to generate a triplet diradical intermediate, either by nitrogen abstraction or via a [2+2] cycloaddition. We focus in particular on the next step of the reaction, in which the triplet diradical interconverts to a singlet product. Owing to their rigid structures, these cyclic compounds (or substituted versions of them) are used in pharmaceutical chemistry, natural product synthesis and drug synthesis [38, 39, 40, 41, 42]. The ring strain imparts high C–H bond strength resulting in remarkable kinetic stability [38]. In recent years, there has been significant interest in the synthesis of these molecules [43, 44, 45] in the field of organic chemistry as well as medicinal chemistry, prompting the rise of the term “escape from flatland” [46, 47, 48], as three‐dimensional molecular scaffolds such as housane, ladderane, and cubane serve as biologically active isosteres of “flat” aromatic systems [38, 49, 50]. Additionally, the high kinetic stability of cubanes has even led them to be proposed as candidates for energy storage [51].

**FIGURE 1:**
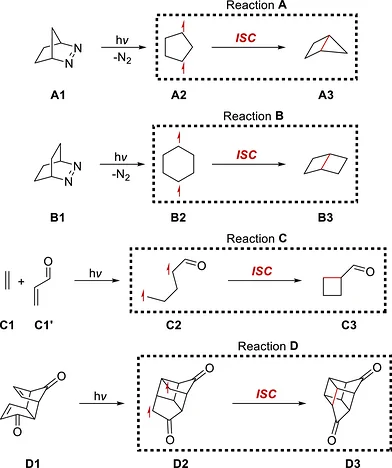
Spin‐forbidden ring closing of triplet diradical intermediates leading to strained singlets.

The ring closing in these diradicals occurs via intersystem crossing (ISC) from a triplet to a singlet state [32, 33, 34, 36, 52, 53], which due to ring strain results in narrow barriers that favor tunneling. This process cannot be explained by conventional transition‐state theory, which assumes a single Born–Oppenheimer PES. Instead, theoretical studies of these nonadiabatic reactions typically locate a minimum energy crossing point (MECP) between the two spin states and utilize nonadiabatic transition state theory (NA‐TST) [54, 55, 56, 57, 58, 59] to calculate the rate. However, this does not account for tunneling effects. The nonadiabatic counterpart for Bell's parabolic‐barrier tunneling correction is the weak‐coupling (WC) approximation, which assumes that the tunneling occurs in one dimension on linear potentials which cross at the MECP [58, 60]. Although this simple approximation is clearly not valid for describing deep tunneling at cryogenic temperatures, it has been assumed to be reliable at high temperatures where the molecule tunnels close to the MECP. However, in this paper we will also show that, even at room temperature, the tunneling pathways are sufficiently extended so as to probe nonlinear parts of the potential, causing the WC approximation to artificially exaggerate the tunneling effects.

For an accurate description of tunneling, one needs to account for the true shape of the PESs as well as multidimensional corner‐cutting effects [61], which allow the molecule to tunnel through a higher but narrower barrier instead of following the minimum‐energy path through the transition state (here represented by the MECP). A method which can reliably capture these effects is nonadiabatic instanton theory [62]. It provides a way to locate the optimal tunneling pathway in full dimensionality, thereby capturing the nonlinearity of the PESs generated by ab initio electronic‐structure theory. Using these results, we will show that heavy‐atom tunneling dominates the mechanism of two of the four reactions studied, even at room temperature.

## 2 Results and Discussion

Before tackling the larger systems, we first study reaction **A**: the ring closing of cyclopentane‐1,3‐diyl (**A2**) leading to the formation of housane (**A3**) [32]. In addition to being the smallest of the four molecules, making it easier to treat with electronic‐structure methods, there are experimental measurements of the rate that can be used to benchmark our theoretical methodology.

Sarkar et al. [33] generated the triplet diradical **A2** by photolysis of **A1** in matrix isolation at low temperatures (5–20 K). They then used electron paramagnetic resonance and infrared spectroscopy to measure the rate at which the triplet diradical underwent ISC to form singlet **A3**. The observed temperature independence of the rate provided evidence in favor of heavy‐atom tunneling. However, the experimental data could not be reproduced by their theoretical calculations [33]. In fact, the authors explicitly highlighted the lack of reliable theoretical methods to compute tunneling effects for a process involving an ISC and were thus forced to use a theory appropriate only for adiabatic reactions. An alternative attempt to compute this rate within a nonadiabatic framework was based on the WC approximation [52]. However, the WC method is known to fail at low temperature, due to the linear approximation of the potentials around the MECP, which is not representative of the true PESs [63, 64]. This explains why the previously reported results significantly overestimated the experimentally measured rate [52]. In this work, we will revisit this problem using the more advanced and newly developed nonadiabatic instanton theory [62].

Before introducing nonadiabatic instanton theory, we first outline the NA‐TST and WC approximations. The NA‐TST rate constant is defined as [54, 55, 56, 57, 58, 59]

(1) $$k_{\text{NA-TST}}=\sqrt{\frac{2\pi m}{\beta \hbar^{2}}}\frac{\Delta^{2}}{\hbar |g_{R}-g_{P}|}\frac{Z^{‡}}{Z_{R}}\;e^{-\beta V^{‡}}$$

where $V^{‡}$ is the energy of the MECP relative to the reactant minimum and Δ is the spin–orbit coupling measured at the MECP. Here, $g_{R/P}$ are the signed norms of the mass‐weighted gradients of the reactant and product surfaces at the MECP. All the reactions were found to have cusped (rather than sloped) crossings, where $g_{R}$ and $g_{P}$ have opposite signs. Because we use mass‐weighted gradients, m is set to a unit mass. Finally, $Z_{R}$ and $Z^{‡}$ are the partition functions of the reactant and MECP, each of which can be decomposed into a product of translational, rotational, and vibrational contributions. In addition, $Z_{R}$ includes a factor of 3 to account for the spin‐degeneracy of the triplet reactant. From the exponent, it is clear that NA‐TST has the form of a classical transition‐state theory which neglects tunneling and will therefore predict an Arrhenius dependence on the temperature.

In order to apply NA‐TST, we located the MECP between the singlet and triplet surfaces and calculated the gradients and Hessians of each state. For these calculations, we used density functional theory (DFT) with the hybrid B3LYP functional and the def2‐TZVPPD basis as implemented in the ORCA program [65].

The WC correction accounts for tunneling effects in an approximate manner. It is derived using a quantum‐mechanical treatment of a simplified system of two crossed one‐dimensional linear potentials in the weak‐coupling limit (Δ→0) [60]. In the high‐temperature regime (where we can ignore the effects of a lower bound on the energy),^2^ the WC correction to the NA‐TST rate constant can be written as [59, 64]

(2) $$k_{\mathrm{WC}}=k_{\text{NA-TST}}\;e^{{\left(T_{o}/T\right)}^{3}}$$

where

(3) $$T_{o}^{3}=\frac{\hbar^{2}}{24mk_{B}^{3}}{\left(\frac{g_{R}g_{p}}{g_{R}-g_{P}}\right)}^{2}$$

Therefore, according to the WC approximation, the tunneling factor, $\kappa_{\text{WC}}=k_{\text{WC}}/k_{\text{NA-TST}}$, depends only on the ratio between the temperature and the parameter $T_{o}$, which is determined by the shape of the cusp. In this way, $T_{o}$ can be interpreted as an “onset temperature” below which tunneling effects become important [64]. In particular, only when $T≲T_{o}$ will tunneling dominate the mechanism and more than double the rate. For reaction **A**, the onset temperature was determined to be 389 K, implying that tunneling increases the reaction rate at room temperature by almost an order of magnitude. However, this estimate is based on the WC approximation, which, as we have already argued, is certainly not reliable at low temperature and, as we will show, may not even be reliable at room temperature.

To go beyond the WC approximation, we employ nonadiabatic instanton theory, which accounts for multidimensional tunneling through a rigorous semiclassical approach. Instanton theory has become a well‐established method for simulating tunneling in molecules [66, 67], since it exhibits an excellent balance between accuracy and computational efficiency. Although it was originally developed for adiabatic reactions [61], it has recently been generalized to nonadiabatic processes [59, 62], where it has achieved excellent agreement with experimental results [63, 64, 68, 69]. Instanton theory is based on the path‐integral formulation of quantum mechanics [70] and locates the optimal tunneling path from the reactant (triplet) to the product (singlet) states using the principle of least action.

In practice, we discretize the instanton path into a ring polymer [59] and search for a first‐order saddle point based on potentials, gradients, and Hessians calculated on‐the‐fly using the same DFT as for the MECP calculations. We optimize instanton pathways at various temperatures and find that for all of the diradicals, the carbon atoms showed considerable delocalization during the reaction, making this a heavy‐atom tunneling process. In fact, the mass‐weighted squared path length shows that the carbon atoms contribute more than 75% of the tunneling pathway at 300 K.

The instanton expression for the rate constant is [59]

(4) $$k_{\mathrm{inst}}=\sqrt{2\pi }\frac{\beta \Delta^{2}}{\hbar }\frac{Z^{‡}}{Z_{R}}e^{-S/\hbar }$$

where S is the action of the tunneling pathway and $Z^{‡}$ is the instanton partition function, which additionally includes fluctuations along the reaction coordinate. The expression constitutes a generalization of the NA‐TST rate in Equation (1) to include tunneling, the key difference being that the activation energy in the exponent is replaced by the action. As $S/\hbar <\beta V^{‡}$, this enhances the rate and causes a deviation from Arrhenius behavior. The instanton tunneling factor is defined as $\kappa_{\text{inst}}=k_{\text{inst}}/k_{\text{NA-TST}}$.

Compared with the WC approximation ($\kappa_{\text{WC}}=8.85$), instanton theory gives a lower tunneling factor ($\kappa_{\text{inst}}=4.20$), although this still suggests a significant increase in the rate due to tunneling at room temperature. However, before using this result to confirm our hypothesis, we should benchmark the theory against experiment. Figure 2 shows that although the calculated instanton rate constants follow a physical behavior (Arrhenius at high temperatures and plateauing at low temperatures [71]), they reach a low‐temperature limit which is orders of magnitude slower than the experimental measurement. We attribute the main cause of this discrepancy to errors in the electronic‐structure calculations, as DFT is known to predict singlet–triplet gaps inaccurately due to the approximate form of the exchange–correlation functional [72]. To counteract this error, we shifted the singlet energy computed by DFT down relative to the triplet. We defined this shift to be the difference between the triplet and singlet energies calculated using multireference methods (CASPT2, complete active space perturbation theory) [73] at the MECP geometry previously optimized using DFT. After applying the correction, the MECPs were reoptimized leading to the significantly reduced barrier heights presented in Table 1.

**TABLE 1:** Barrier heights, $V^{‡}$, (defined as the energy difference between the MECP and the minimum) and onset temperatures, $T_{o}$, [Equation (3)] calculated for the ring‐closing process in the four reactions (before and after applying the CASPT2 shifts).

Reaction	$V^{‡}$ (kJ ${\mathrm{mol}}^{-1}$)	$T_{o}$ (K)	shift (kJ ${\mathrm{mol}}^{-1}$)	$V_{\mathrm{shift}}^{‡}$ (kJ ${\mathrm{mol}}^{-1}$)	$T_{o}$ 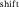 (K)
**A**	46.2	389	41.2	29.0	348
**B**	55.6	357	41.8	29.8	287
**C**	31.2	351	42.7	14.6	279
**D**	30.7	389	25.8	19.6	361

**FIGURE 2:**
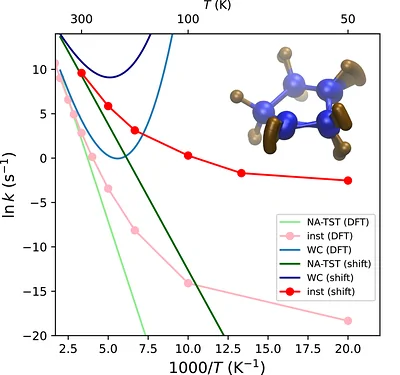
Thermal rate constants for reaction **A** computed using NA‐TST, the WC approximation, and nonadiabatic instanton theory. The instanton results plateau to a low‐temperature limit, while the WC approximation breaks down at low temperatures.

After including the CASPT2 shift, the instanton rates are substantially increased, as shown in Figure 2. In particular, the calculated low‐temperature rate constant is corrected from $3.7\times 10^{-7}$ to $0.1\;s^{-1}$. Another iteration of the procedure gives a further CASPT2 shift of 11.5 kJ ${\mathrm{mol}}^{-1}$, which increases the rate constant (see Supporting Information) to 0.6 $s^{-1}$, bringing it into reasonable agreement with the low‐temperature experimental result, reported as 2.48 or 2.71 $s^{-1}$ from measurements at 5 and 20 K [33]. This resolves the order‐of‐magnitude discrepancy between theory [52] and experiment [33] and validates our theoretical approach.

The shifted calculations also give corrected values for the onset temperatures, as presented in Table 1. There is only a small decrease, implying that the barrier shape is reasonably well described by DFT even if the barrier height was too high. Instanton calculations confirmed that the tunneling contribution to the reaction rate remains significant at room temperature (albeit with a slightly smaller tunneling factor of $\kappa_{\text{inst}}^{\text{shift}}=2.7$). These results imply that existing housane synthesis strategies, which predominantly depend on radical pathways [33, 44, 74], are taking place via room‐temperature heavy‐atom tunneling. In fact, the major reason for the significance of the housane‐motif in asymmetric synthesis and drug design is due to its non‐planar structure. The instanton pathway shows that tunneling assists the key step, in which an almost planar triplet is converted to a non‐planar singlet.

At this point, we investigate why there is such a large discrepancy between instanton theory and the WC approximation. It is of course clear that the WC approximation fails at low temperatures, where the potentials along the deep‐tunneling pathway can certainly not be well represented by linear functions. However, one might have assumed it to be a reasonable approximation at high temperatures, where the tunneling pathway shrinks close to the MECP.

One reason for the discrepancy is simply that the potentials along the tunneling pathway are not well described by linear functions, even over the short distance explored. Although in Figure 3a only a small amount of curvature is apparent, it is clear that the barrier is wider than assumed by the WC approximation, leading to smaller tunneling factors. Additionally, instanton theory can employ corner‐cutting effects to distort the tunneling pathways around the MECP so as to minimize the barrier width. However, as is evident from Figure 3b, this effect only becomes relevant as we decrease the temperature.

**FIGURE 3:**
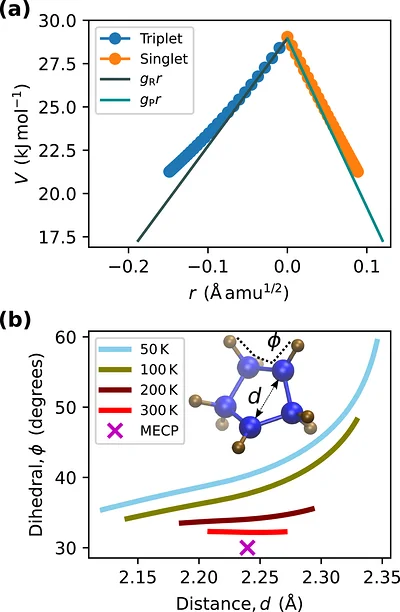
(a) Potentials along the mass‐weighted reaction coordinate, r, according to instanton theory (circles) and the WC linear approximation (lines) for reaction **A** at 300 K (both using the CASPT2 shift). (b) Corner cutting of the instanton pathways at various temperatures plotted against the C–C distance and the H–C–C–H dihedral angle indicated in the inset.

Importantly, our conclusions on the role of heavy‐atom tunneling at room temperature is not limited to the housane reaction. In fact, we assume it will be a common occurrence for spin‐forbidden reactions of strained organic molecules. This hypothesis is confirmed by additionally computing instantons for the ring closing of reactions **B**–**D**, which are shown in Figure 4.

**FIGURE 4:**
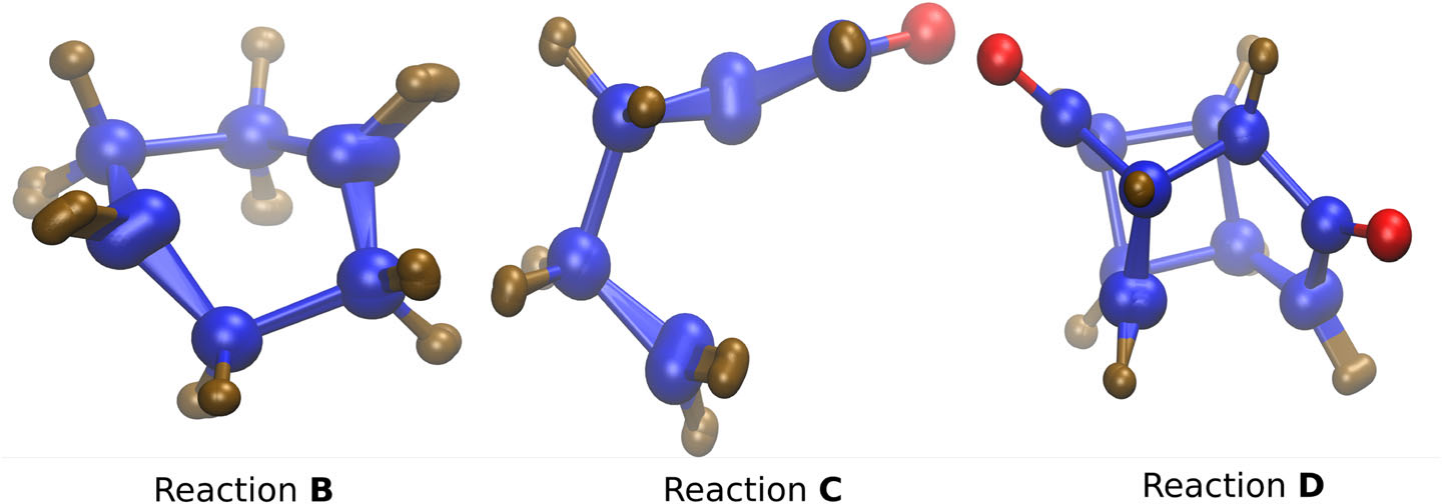
Heavy‐atom tunneling accompanying ISC in the ring‐closing reactions for the larger strained molecules. The images show the instanton trajectories (calculated without the CASPT2 shifts) for each of the reactions at 75 K. The pathways at 300 K (with or without the shifts) are similar but with a smaller amount of delocalization.

In each case, the results presented in Table 2 show a similar behavior: the estimate from WC/DFT is too high and the tunneling factor is systematically lowered by including the CASPT2 correction and using the more accurate instanton theory. Nonetheless, even after these corrections, it is clear that tunneling has a role to play in all four reactions by reducing S/ℏ relative to $\beta V^{‡}$ (as indicated by $\kappa_{\text{1D}}^{\text{shift}}$). However, the effect on reactions **A** and **D** is significantly stronger than for reactions **B** and **C**. This is probably a consequence of the increased ring strain, leading to a narrower barrier. In particular, reaction **D** has a tunneling factor of $\kappa_{\text{inst}}^{\text{shift}}=2.6$, and is thus dominated by heavy‐atom tunneling similar to reaction **A**. In contrast, the less strained reactions **B** and **C** are sped up by less than a factor of 2, such that the over‐the‐barrier process dominates. This observation suggests that the propensity of heavy‐atom tunneling is higher for spin‐forbidden reactions of strained diradicals.

**TABLE 2:** Tunneling factors at 300 K obtained from the WC approximation, 1D approximation (defined as $\kappa_{\text{1D}}=e^{-S/\hbar }/e^{-\beta V^{‡}}$) and nonadiabatic instanton theory. The results are shown both before and after applying the CASPT2 shift.

Reaction	$\kappa_{\mathrm{WC}}$	$\kappa_{\text{1D}}$	$\kappa_{\mathrm{inst}}$	$\kappa_{\mathrm{WC}}^{\mathrm{shift}}$	$\kappa_{\text{1D}}^{\mathrm{shift}}$	$\kappa_{\mathrm{inst}}^{\mathrm{shift}}$
**A**	8.9	5.7	4.2	4.8	3.3	2.7
**B**	5.4	4.2	2.7	2.4	1.8	0.5
**C**	5.0	3.5	2.9	2.2	1.8	1.6
**D**	8.9	5.4	3.7	5.7	3.6	2.6

The calculated results for reaction **B** give a tunneling factor of $\kappa_{\text{inst}}^{\text{shift}}<1$, which appears to suggest that tunneling actually slows down the rate. This is, however, not the case. The reason for the result is that the instanton explores strained configurations with particularly stiff bonds, where the zero‐point energy correction raises the effective barrier height. NA‐TST only has information from the MECP, and thus does not correctly describe the changing frequencies along the reaction coordinate. A variational version of NA‐TST (similar to that used in adiabatic rate theories [75]) would give a lower rate, leading to a value of $\kappa_{\text{inst}}^{\text{shift}}$ larger than 1. This argument may also affect the other three reactions, but as it can only increase the tunneling factor, it will not change our conclusions of the role of tunneling.

There is one more aspect to consider before we can draw conclusions about real experiments from our theoretical simulations. In our calculations, the molecules are modeled in isolation. This may be a good approximation for a weakly interacting Argon matrix [33]; however, most organic synthesis is carried out in solution. To determine the solvent effect on the reactions, we employed a conductor‐like polarizable continuum model (CPCM) [76] to mimic the effects of tetrahydrofuran (which is commonly used in these reactions [44, 50]) and found the barrier height to decrease by only a few kJ ${\mathrm{mol}}^{-1}$ (see Supporting Information), which is smaller than the CASPT2 shift and thus although it may affect the rates by about an order of magnitude, it is not expected to significantly affect the tunneling factors. The weak role played by the solvent in these reactions is probably due to the absence of charge separation. This suggests that our finding of heavy‐atom tunneling in these isolated molecules carries over into chemistry in solution.

Finally, we discuss why the role of heavy‐atom tunneling at room temperature in these reactions has not previously been noticed. The calculated rate constants for the reactions at room temperature are given in Table 3 (along with the calculated spin–orbit couplings, which are required to obtain absolute rate constants rather than just tunneling factors). At these temperatures, the timescales for the reactions are on the order of microseconds to milliseconds. Thus, from the point of view of a synthetic chemist, the reactions are effectively instantaneous and hard to measure without a complicated time‐resolved spectroscopy apparatus. Kinetic isotope effects are commonly used to quantify tunneling effects in hydrogen‐transfer reactions and occasionally to probe carbon and nitrogen tunneling. However, the small relative mass difference between 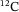 and 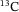 means that only very small KIEs are expected, even in the presence of a factor‐of‐2 tunneling speed‐up. In particular, we calculate tiny KIEs between 1.03 and 1.06 for the four reactions at room temperature when the two radical 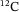 atoms are isotopically substituted by 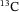 (see Supporting Information). Nonetheless, even though experiments are not easily able to measure such small isotope effects, theoretical approaches, such as the one presented in this work, are able to provide evidence for heavy‐atom tunneling at room temperature by comparing the rates obtained from quantum and classical mechanics.

**TABLE 3:** Spin–orbit couplings, Δ, (calculated at the MECP) and rate constants (calculated by nonadiabatic instanton theory) for the four reactions at 300 K, before and after applying CASPT2 shifts.

Reaction	Δ (${\mathrm{cm}}^{-1}$)	$k_{\mathrm{inst}}\;\left(s^{-1}\right)$	$\Delta_{\mathrm{shift}}$ (${\mathrm{cm}}^{-1}$)	$k_{\mathrm{inst}}^{\mathrm{shift}}\;\left(s^{-1}\right)$
**A**	4.9	$1.7\times 10^{1}$	4.0	$1.5\times 10^{4}$
**B**	1.4	$1.2\times 10^{-2}$	1.7	$7.0\times 10^{2}$
**C**	2.0	$2.2\times 10^{2}$	1.6	$2.7\times 10^{5}$
**D**	5.3	$1.5\times 10^{4}$	4.7	$1.1\times 10^{6}$

## 3 Conclusion

We have studied the spin‐forbidden ring closing of organic diradicals over a wide range of temperatures. We see that heavy‐atom tunneling can dominate the reaction mechanism even at room temperature and that this effect significantly increases as the temperature is lowered. The validity of our theory is demonstrated by the order‐of‐magnitude agreement of the calculated reaction rate constants with the experimental measurements of the formation of housane at low temperature. To achieve this, we employed nonadiabatic instanton theory with on‐the‐fly DFT calculations including a CASPT2 shift to correct the singlet–triplet gap. As the tunneling effect is much greater and more difficult to describe at low temperature, this agreement with experiment also validates our approach at high temperatures.

Our results highlight the shortcomings of traditionally used tunneling corrections such as Bell's formula or the WC approximation even at room temperature. Bell's parabolic approximation is only applicable to adiabatic reactions, whereas the nonadiabatic WC method not only breaks down at low temperature but also significantly overestimates the contribution of tunneling at room temperature. Nonadiabatic instanton theory on the other hand resolves these problems and provides a more rigorous calculation of the rate. Furthermore, instanton theory provides mechanistic insight via the optimal reaction pathway. In future studies, it may be possible to harness the predictive power of instanton theory to identify reactions in which one can attain tunneling control over regioselectivity or stereochemistry while synthesizing strained molecules without even needing to work at cryogenic temperatures.

Our findings indicate that heavy‐atom tunneling plays a more significant role during ring closure of strained organic radicals than has hitherto been presumed. The reasons for tunneling at room temperature in this class of molecules are two fold: first, the ring strain in the molecules causes the reaction barriers to be narrow; second, the barrier top for the nonadiabatic reaction is cusped unlike the rounded top of adiabatic reactions. These conditions are ideal for tunneling. As this scenario is not uncommon, heavy‐atom tunneling at room temperatures may be more prevalent in organic chemistry than previously thought. In fact, it seems that many syntheses of strained molecules reported in the literature [37, 43, 44, 45, 50] were occurring via heavy‐atom tunneling without the knowledge of the chemist working in the laboratory!

## Conflicts of Interest

The authors declare no conflicts of interest.

## Supporting information


**Supporting File 1**: anie74826‐sup‐0001‐SuppMat.pdf.

**Supporting File 2**: anie74826‐sup‐0001‐SuppMat.zip.

## Data Availability

The data that support the findings of this study are available in the supplementary material of this article.

## Endnotes

1 In order to keep the computational cost manageable, we replaced two Br atoms by H atoms.

2 The effect of including a lower bound on the energy, which is the usual practice [58], avoids the unphysical increase in the rate at low temperature observed in Figure 2. However, it is not fundamentally better, as it causes the rate to unphysically decrease [52] instead and gives a result that depends on the arbitrary choice of the lower bound [63].
