# Supplementary material for: Heavy‐Atom Tunneling at Room Temperature Revealed by Instanton Theory
Source: Angew Chem Int Ed Engl. 2026 Sep 23;65(45):e2372199. doi: 10.1002/anie.2372199 (PMC13725619; doi:10.1002/anie.2372199)
Supplement: Supplementary file 1 [file ANIE-65-e2372199-s001.pdf]

Supporting Information

Heavy-Atom Tunneling at Room Temperature Revealed by Instanton Theory

Debaarjun Mukherjee,[+] Kamil D. Petryczkiewicz,[+] Jeremy O. Richardson *

Department of Chemistry and Applied Biosciences, ETH Zürich, 8093 Zürich, Switzerland
*E-mail: jeremy.richardson@phys.chem.ethz.ch

[+] These authors contributed equally.

Contents

1 Computational Details **2**

2 CPCM Model **2**

3 Instanton Optimization **3**

4 Kinetic Isotopic Effect **3**

1 Computational Details

For all four reactions studied, potential energy surfaces were evaluated on-the-fly using density functional theory (DFT) as implemented in the ORCA software package.[1] We chose the B3LYP functional along with the def2-TZVPPD basis set. Unless explicitly mentioned, in all our calculations we have studied isolated molecules. The reactant minimum was optimized on the triplet surface. We located the minimum energy crossing point between the singlet and triplet surfaces and calculated the onset temperatures from the mass-weighted gradients at this geometry.

After locating the MECP, we performed state-specific CASPT2 calculations of both the singlet and triplet states using the same basis-set as before. These CASPT2 calculations were performed using the Molpro software package.[2] The active space selected for each of the reactions are given in Table S1. In each case, CASPT2 predicted the triplet energy to be higher than the singlet energy at the MECP. This energy gap defined the shift used to correct the DFT singlet energies, as presented in the main text.

Table S1. The active spaces used for the CASPT2 calculations defined as (number of electrons, number of orbitals)

Reaction	Active Space
**A**	(10,10)
**B**	(14,10)
**C**	(14,10)
**D**	(16,10)

For reaction A only, we performed a second iteration and calculated the CASPT2 energies at the reoptimized MECP. This gave a further shift of 11.53 $\mathrm{kJ\,mol^{-1}}$. On these shifted surfaces, the MECP barrier height decreased from 28.95 to 28.57 $\mathrm{kJ\,mol^{-1}}$ (i.e. by a factor of 0.99). Instead of reoptimizing the instantons again, a simple correction to the rate was obtained scaling the potentials (relative to the reactant minimum) along the previous instanton trajectory by a factor of 0.99. This decreased the action $S/\hbar$ from 24.07 to 22.02 to give the corrected rate presented in the main text.

2 CPCM Model

We used the conductor-like polarizable continuum model (CPCM),[3] as implemented in ORCA, to simulate solvent effects. Using the MECPs calculated using the DFT potential energy surfaces including the CASPT2 shift (but without solvent effects), we ran single-point calculations using the CPCM model parametrized to describe solvation in tetrahydrofuran (THF). Table S2 presents the new barrier heights, $V^\ddagger$, calculated by evaluating the average between the singlet (after applying the CASPT2 shift) and triplet energies at the MECP geometry and then subtracting the energy of the triplet minimum geometry. It additionally presents the singlet–triplet energy gap caused by solvent effects at the MECP geometry. In general, solvent effects lower the energy barrier, $V^\ddagger$, and open up the MECP by a few $\mathrm{kJ\,mol^{-1}}$. However, the effect is much smaller than the CASPT2 shift that was described in the main text.

Table S2. Singlet–triplet gaps and barrier heights, $V^\ddagger$, in $\mathrm{kJ\,mol^{-1}}$ computed using the CPCM model at the MECP of the molecule in vacuum.

Reaction	$E_\mathrm{T} - E_\mathrm{S}$	$V^\ddagger$
**A**	1.14	27.83
**B**	4.49	26.63
**C**	10.01	8.84
**D**	8.15	15.44

3 Instanton Optimization

In the ring-polymer instanton formalism, the action is defined as

$$S(\boldsymbol{x}, \tau) = \sum_{i=1}^{N_\mathrm{R}} \left(\frac{m||\mathbf{x}_i - \mathbf{x}_{i-1}||^2}{2\epsilon_\mathrm{R}} + \frac{1}{2}\epsilon_\mathrm{R} \left[V_\mathrm{R}(\mathbf{x}_i) + V_\mathrm{R}(\mathbf{x}_{i-1}) \right] \right) + \sum_{i=N_\mathrm{R}+1}^{N} \left(\frac{m||\mathbf{x}_i - \mathbf{x}_{i-1}||^2}{2\epsilon_\mathrm{P}} + \frac{1}{2}\epsilon_\mathrm{P} \left[V_\mathrm{P}(\mathbf{x}_i) + V_\mathrm{P}(\mathbf{x}_{i-1}) \right] \right)$$

where $\boldsymbol{x} = \{\mathbf{x}_1, \mathbf{x}_2, \dots, \mathbf{x}_N\}$ is the combined vector of all the bead positions (with cyclic indexing $\mathbf{x}_0 \equiv \mathbf{x}_N$) and $\epsilon_n = \tau_n/N_n$ for $n \in \{\mathrm{R}, \mathrm{P}\}$. Note that the imaginary time is split into two parts: $\tau_\mathrm{P} = \tau$ on the product and $\tau_\mathrm{R} = \beta\hbar - \tau$ on the reactant, where $\beta = 1/k_\mathrm{B}T$ with T being the temperature and k_B the Boltzmann constant. Instantons were optimized by searching for a first-order saddle point of the action S in position, $\boldsymbol{x}$, and imaginary-time, τ, coordinates.

The final instantons at 300 K were optimized using $N = 128$ beads. For housane at lower temperatures, 256 and 512 beads were used for 75 K and 50 K, respectively. In each case, the reactant and product beads were evenly split such that $N_\mathrm{R} = N_\mathrm{P} = N/2$. The instantons were optimized starting from above the onset temperature, where the instanton is practically collapsed at the MECP. Consequently, the instantons were cooled using the previous instanton as a starting guess for the next one. We optimized instantons for both the bare DFT surfaces as well as the surfaces obtained after adding the CASPT2 shift. The converged instantons are provided are supplementary data files.

The action S as well as $\beta V^\ddagger$ for each of the reactions before and after the shift at 300 K are given in Table S3. We see that at 300 K, the action is lower than $\beta V^\ddagger$, which highlights the role of tunneling at room temperature.

Table S3. The action S (in units of $\hbar$) is compared with $\beta V^\ddagger$ for each of the reactions (before and after the CASPT2 shift) at 300 K, highlighting the speed-up due to tunneling.

Reaction	$\beta V^\ddagger$	$S/\hbar$	$\beta V^\ddagger_\mathrm{shift}$	$S_\mathrm{shift}/\hbar$
**A**	18.50	16.76	11.61	10.42
**B**	22.29	20.85	11.93	11.32
**C**	12.52	11.25	5.86	5.27
**D**	12.30	10.61	7.87	6.60

4 Kinetic Isotopic Effect

After obtaining the instanton at a particular temperature, we can estimate the kinetic isotope effect (KIE) using a simple approximation. Using the same tunneling pathway, we isotopically substitute certain atoms of interest and recalculate S. Since the rate is proportional to $\mathrm{e}^{-S/\hbar}$ with a prefactor that is roughly constant, the KIE is to a first approximation given by $\mathrm{e}^{-(S_\mathrm{new} - S_\mathrm{old})/\hbar}$.

To understand the role of tunneling compared with that of zero-point energy, we also calculate the KIE effect from NATST by calculating the rates after isotopically substituting the atoms of interest. As seen from Tables S4 and S5, there is a minor KIE due the carbon isotope substitution, mostly due to the tunneling effect.

References

[1] F. Neese, *WIREs Comput. Mol. Sci.* **2022**, *12*, e1606.

[2] H.-J. Werner, P. J. Knowles, F. R. Manby, J. A. Black, K. Doll, A. Heßel-

Table S4. KIEs estimated from nonadiabatic instanton theory

Reaction	$S_{\mathrm{old}}/\hbar$	$S_{\mathrm{new}}/\hbar$	$\mathrm{KIE}_{\mathrm{inst}}$
**A**	10.42	10.47	1.04
**B**	11.32	11.35	1.03
**C**	5.27	5.30	1.03
**D**	6.60	6.66	1.06

Table S5. KIEs computed from NA-TST

Reaction	$k^{\mathrm{old}}_{\mathrm{NA\text{-}TST}}$ (s^{-1})	$k^{\mathrm{new}}_{\mathrm{NA\text{-}TST}}$ (s^{-1})	$\mathrm{KIE}_{\mathrm{NA\text{-}TST}}$
**A**	5.43×10^3	5.39×10^3	1.008
**B**	1.45×10^3	1.49×10^3	0.975
**C**	1.66×10^5	1.67×10^5	0.996
**D**	4.09×10^5	4.06×10^5	1.005

mann, D. Kats, A. Köhn, T. Korona, D. A. Kreplin, et al., *J. Chem. Phys.* **2020**, *152*.

[3] V. Barone, M. Cossi, *J. Phys. Chem. A* **1998**, *102*, 1995.
